# Biomass Allocation and Leaf Morphology of Saplings Grown under Various Conditions of Light Availability and Competition Types

**DOI:** 10.3390/plants11030305

**Published:** 2022-01-24

**Authors:** Ieva Bebre, Isa Marques, Peter Annighöfer

**Affiliations:** 1 Spatial Structures and Digitization of Forests, Faculty of Forest Sciences and Forest Ecology, University of Göttingen, Büsgenweg 1, 37077 Göttingen, Germany; 2 Spatial Data Science and Statistical Learning, Faculty of Business and Economics, University of Göttingen, Platz der Göttinger Sieben 3, 37073 Göttingen, Germany; imarques@uni-goettingen.de; 3 Forest and Agroforest Systems, School of Life Sciences, Technical University of Munich (TUM), Hans-Carl-von-Carlowitz-Platz 2, 85354 Freising, Germany; peter.annighoefer@tum.de

**Keywords:** controlled experiment, pots, specific leaf area, quantile regression

## Abstract

Plant growth is almost always limited by light availability and competition. However, plants are generally plastic and can change their morphology and biomass allocation to optimize growth under suboptimal conditions. We set up a controlled pot experiment with three light availability levels (10%, 20%, and 50%) to study the effect of light and competition on the biomass allocation and leaf morphology in monospecific and mixed pots of recently planted European beech (*Fagus sylvatica* L.), Norway spruce (*Picea abies* (L.) Karst.), and Douglas fir (*Pseudotsuga menziesii* (Mirb.) Franco) saplings using a quantile regression model. Specific leaf area (SLA) showed the strongest reaction and increased with decreasing light availability. Woody aboveground mass fraction (AMF) increased with decreasing light availability, but the effect of light on biomass allocation was less pronounced than on SLA. The SLA, woody AMF, and root mass fraction (RMF) of the two conifer species and European beech varied greatly, with European beech having a higher SLA and RMF than the two conifer species. The associated effect of plant size on biomass allocation was small, and the strength of the association was not meaningful on a practical level. The competitor’s effect on biomass allocation was minor overall and only present for some species, suggesting that species’ functional dissimilarity does not greatly affect allocational patterns in early tree development stages.

## 1. Introduction

Plant growth is almost always limited by resource availability, competition, herbivores, and other factors that make the growing conditions suboptimal [1]. Plants grow when the carbohydrates manufactured in leaves are assimilated into new cells [2]. Growth is a crucial element of plant fitness and determines the plant’s size and the outcome of competitive interactions [3]. Each plant organ has a specific function in resource acquisition, the provision of assimilate pathways, and mechanical support [4,5]. Moreover, plants can exhibit plasticity and adjust the distribution of organs and their systems in response to available resources [6].

Plasticity is a desirable species characteristic that varies interspecifically, with ontogeny, and is a particularly crucial trait for sessile organisms since they cannot escape the unfavorable growing conditions [7,8]. Plastic species can change their morphology, architecture, and biomass allocation to optimize growth and better the chances of survival in suboptimal conditions [9]. In addition to species-specific plasticity, biomass allocation patterns change with ontogeny and become less flexible as plants develop [6,10]. The higher the species plasticity, the faster the plant can react to changes in resource availability; however, this behavior can also be futile and result in higher mortality due to growth resource exhaustion [8]. Therefore, it is essential that the plastic response is adaptive and increases plants’ fitness [11].

The *balanced growth hypothesis* describes changes in biomass allocation depending on resource availability and states that a plant will allocate its biomass towards the limiting resource [12]. The balanced growth hypothesis belongs to the optimal partitioning theory, suggesting trade-off-based flexibility in biomass allocation between plant organs to optimize resource acquisition when their availability is limited [13,14]. This theory has been challenged by the allometric partitioning theory, which, on the other hand, suggests that biomass allocation is scaled to and constrained by a plant’s size [6,15]. The two theories can, however, also be viewed as complementary. There is already scientific evidence suggesting that both allometric and optimal partitioning coincide [15,16,17]. For example, when studying biomass allocation at different light and water availability levels, Schall et al. [18] found that biomass partitioning between the above and belowground organs of young trees was influenced by the growing conditions (optimal partitioning). In contrast, the partitioning between aboveground organs was size-dependent (allometric partitioning).

Light is one of the most crucial resources determining plant survival and growth. How well species can tolerate or are adaptable to low light environments affects their ability to regenerate, persist, and grow in the shade [19,20,21]. The effect of light on tree growth is so central that the species are often classified based on their light requirements, e.g., pioneer and non-pioneer species, shade-tolerant and light-demanding species, and gap-specialists [21]. According to the balanced growth hypothesis, if the light availability is limited, the plants will allocate their growth towards the aboveground compartments either directly (leaves/needles) or indirectly (branches and stems) responsible for light uptake. Increased biomass allocation to one organ changes the overall biomass partitioning patterns between the organs as plants respond to the growing conditions and optimize their ability to uptake limited resources [22].

Leaves are plastic organs, and plants tend to adapt to limited light availability by firstly increasing biomass allocation to leaves and changing their morphology [5,23]. One of the most common and fastest phenotypic adaptations to shade is increasing specific leaf area (SLA). Namely, plants produce thinner or larger leaves/needles, thus increasing the photosynthetic surface area [24]. Plasticity in SLA is generally greater than in leaf mass fraction (LMF), and plants in low light environments are more likely to increase the photosynthetic rate per leaf surface by increasing SLA (morphological changes) than by allocating more biomass to leaves or needles (allocational changes) [5].

Plants can improve their chances for more light acquisition through allocational changes in the woody aboveground compartments. Stem elongation is a common strategy for more light-demanding species that attempt to maintain contact with a light source through vertical growth [25,26]. Biomass allocation to branches depends on multiple factors and is not limited only by light availability but also by stand or regeneration density and the proximity of direct competitors [27]. Additionally, there are pronounced genetic differences between branching patterns among broadleaf and conifer species, with conifers having a stronger apical dominance and pronounced branch whorls [28,29].

When growth is limited by light availability, biomass allocation to roots is decreased. However, when the light availability is high, plants invest more in root mass to cope with high radiation loads and compensate for the high transpiration losses through increased water uptake [30].

Under field conditions, plant growth is rarely restricted by only one limiting resource. Moreover, the growth and biomass allocation depend not only on the availability of resources and the presence of various stressors but also on the species-specific response and competitive conditions [18]. Two processes that comprise the community’s net competitive effect on tree performance are the community’s effect on resource availability and the species-specific response to reduced resource availability [31,32]. When a resource, such as light, is limited, the competition can occur on two levels: directly—through encroaching between the plants—and indirectly—through the indirect exploitation of a resource [33]. Assuming a size advantage, species that can tolerate low-resource environments will be dominant in competitive situations [32].

The community effect on tree growth has been intensely studied for mixed forests. Most studies looking at tree performance in terms of timber volume or biomass have agreed that factors like niche complementarity and species’ functional dissimilarity (here, phenotypically, phyletically, and functionally different species) play a crucial role in how the community structure influences tree development [34,35]. However, other factors like plant size and ontogeny also influence allocation patterns [5]. Studies examining mixing effects have found that European beech (*Fagus sylvatica* L.) performs better in mixtures than in monospecific stands due to its low self-tolerance, whereas the productivity of Norway spruce (*Picea abies* (L.) Karst.) depends on resource facilitation and site quality [36,37]. The growth of Douglas fir (*Pseudotsuga menziesii* (Mirb.) Franco), on the other hand, is greatly limited by light availability, but when that is sufficient, the species is highly competitive [38,39].

Forestry practices in Germany are leaning towards abstaining from large clear-cuts, which—in the long term—means that the next generation of trees will grow under cover of older generations and, thus, under conditions of limited light availability [40]. Consequently, ensuring a species mixture of both shade-tolerant and light-demanding species would require that the resource availability under the canopy, mainly light, is sufficient for various species to grow and develop. Therefore, improving the understanding of (1) species-specific responses to resource availability and (2) the effects of a competitor’s type along a light gradient for trees in early development stages is crucial for the management of mixed-species regenerations and should be systematically investigated [12,41].

We set up a controlled pot experiment with European beech, Norway spruce, and Douglas fir saplings. European beech and Norway spruce are economically significant species in Germany, and Douglas fir is considered a more drought-resistant species that could potentially replace Norway spruce as the climate changes and the frequency of drought events increases [42]. The conifer admixture is often seen to increase the functionality of European beech forests and, therefore, is desirable [43]. We studied the effects of light availability and a competitive situation on the biomass allocation and leaf morphology of saplings grown in monospecific and mixed pots. We set up the following hypotheses:

**Hypothesis** **1** **(H1).** *LMF and SLA increase with decreasing light availability for all studied species.*

**Hypothesis** **2** **(H2).** *Woody aboveground mass fraction (AMF) increases with decreasing light availability.*

**Hypothesis** **3** **(H3).** *Biomass allocation patterns change with the sapling size (height and diameter) and are different between coniferous (Norway spruce and Douglas fir) and broadleaved European beech saplings, with conifers allocating more biomass to aboveground than European beech.*

**Hypothesis** **4** **(H4).** *Individuals competing with functionally dissimilar species allocate more biomass to leaf, woody aboveground, and root biomass fractions than individuals competing with functionally similar species due to lower competition pressure.*

## 2. Results

### 2.1. Specific Leaf Area and Leaf Mass Fraction

**Figure 1:**
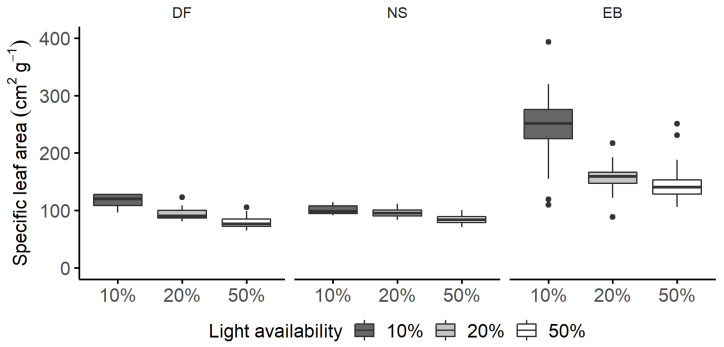
Specific leaf area (SLA, cm^2^ g^−1^) of Douglas fir (DF), Norway spruce (NS), and European beech (EB) saplings grown under 10%, 20%, and 50% light availability levels. Boxplots are based on raw data and graphically depict the SLA dataset. Boxplots are not an outcome of the quantile regression models.

**Figure 2:**
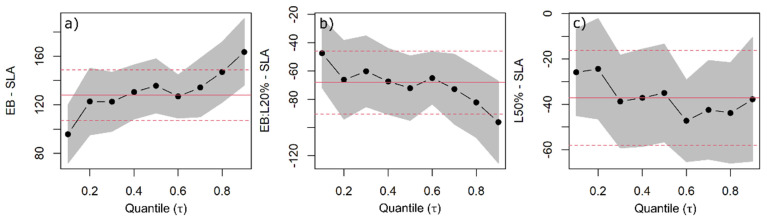
Quantile regression (QR) was used to estimate changes in specific leaf area (SLA) as a function of species, light availability, and competitors’ functional similarity/dissimilarity. QR coefficients of European beech (EB) (**a**), EB interaction with 20% light availability levels (**b**), and at 50% light availability level common to all species (**c**) are shown with the black dots indicating the estimated coefficient for each quantile and the black dotted line indicating the trend with increasing quantiles. The grey band around the dotted line represents the 95% confidence interval for the coefficients. The solid red line is the ordinary least squares (OLS) regression coefficient, and the red dashed lines represent the 95% confidence interval based on OLS.

For 10% light availability and a similar competitor, European beech had a median SLA that was 136 ± 16 cm^2^ g^−1^ higher than for Douglas fir (112 ± 15 cm^2^ g^−1^), whereas the difference between the median SLA for Douglas fir and Norway spruce was insignificant (Figure 1 and Figure 2a, Supplement S9).

Overall, the SLA decreased with increasing light availability for all species (Figure 1, Table S1). The effect of increasing light availability for Douglas fir was only significant at the 50% level, and the decrease amounted to 77 cm^2^ g^−1^ (Figure 2c). We found no significant difference between the response of Douglas fir and Norway spruce to changing light availability. European beech showed the strongest response to increasing light availability (Figure 2b). Namely, the decrease in SLA was significant for both 20% and 50% light availability levels (Figure 2c), with an approximately two-fold increases in the coefficients between the lower 0.1 and the upper 0.9 quantiles. The species’ functional group determined the SLA more than the species’ shade tolerance, with the two conifers behaving similarly towards changing light availability.

Under a 10% light availability level and similar competitor, Norway spruce allocated the most biomass to LMF (21%) compared to Douglas fir (18%) and European beech (14%), and the same ranking was true under a 50% light availability level (Table S2). Only Norway spruce tended to decrease its LMF with increasing light availability; however, the total decrease amounted to only 2%, and it was no longer significant for more extreme quantiles (10% and 90%) under 20% and 50% light availability levels (Table S2).

**Figure 3:**
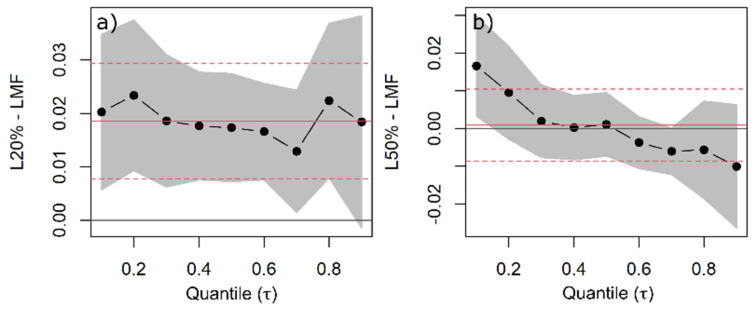
Quantile regression (QR) was used to estimate changes in leaf mass fraction (LMF) as a function of species, light availability, and competitors’ functional similarity/dissimilarity. QR coefficients of 20% (**a**) and 50% (**b**) light availability levels common to all species are shown with the black dots indicating the estimated coefficient for each quantile and the black dotted line indicating the trend with increasing quantiles. The grey band around the dotted line represents the 95% confidence interval for the coefficients. The solid red line is the ordinary least squares (OLS) regression coefficient, and the red dashed lines represent the 95% confidence interval based on OLS.

The LMF of Douglas fir and European beech peaked at 20% light availability. Increasing light availability from 10% to 20% increased the median LMF of Douglas fir by 2% (Figure 3a) and the LMF of European beech by 6% (Table S2). However, further increases in light availability were, overall, insignificant for Douglas fir (Figure 3b) and resulted in only minor increases (by 2%) of the LMF of European beech (Table S2). For Norway spruce, an increase in light availability was associated overall with decreases in LMF. Namely, increases from 10% to 50% were associated with a decrease of about 2% of the LMF Norway spruce.

The effect of sapling size (height and diameter) on biomass allocation to LMF was statistically significant; however, at the median, a 1 cm increase in height was associated with a −0.03% decrease in LMF, and a 1 mm increase in diameter with a 0.1% increase in LMF (Table S5). Due to the small associated effect size, the height and diameter variables were considered not meaningful in explaining biomass allocation to leaves or needles, excluded from the model, and not discussed further.

The differences in LMF were more species-specific and less dependent on species’ functional groups (Table S2). For SLA, the opposite behavior was observed (Table S1).

### 2.2. Woody Aboveground and Root Mass Fractions

**Figure 4:**
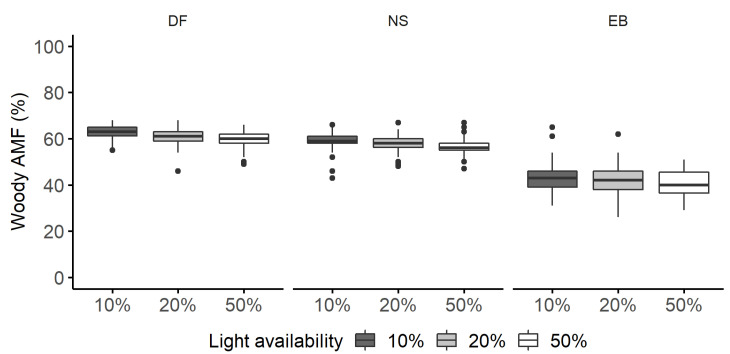
Woody aboveground biomass fraction (AMF, %) of Douglas fir (DF), Norway spruce (NS), and European beech (EB) saplings grown under 10%, 20%, and 50% light availability levels. Boxplots are based on raw data and graphically depict the woody AMF dataset. Boxplots are not an outcome of the quantile regression models.

**Figure 5:**
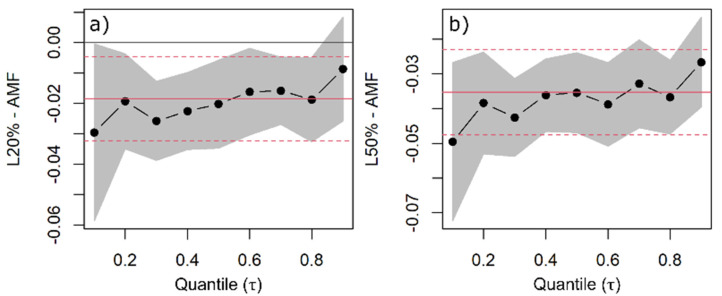
Quantile regression (QR) was used to estimate changes in woody aboveground mass fraction (AMF) as a function of species, light availability, and competitors’ functional similarity/dissimilarity. QR coefficients of 20% (**a**) and 50% light availability levels (**b**)—common to all species—are shown with the black dots indicating the estimated coefficient for each quantile and the line indicating the trend with increasing quantiles. The grey band around the dotted line represents the 95% confidence interval for the coefficients. The solid red line is the ordinary least squares (OLS) regression coefficient, and the red dashed lines represent the 95% confidence interval based on OLS.

There were pronounced differences in the woody AMF of the coniferous and European beech saplings. For 10% light availability and a similar competitor, European beech allocated 16% and 20% less biomass—on the median—to woody aboveground compartments than Norway spruce and Douglas fir, respectively (Figure 4). All species decreased their woody AMF with increasing light availability. The median woody AMF decreased by 2% between 10% and 20% light availability levels (Figure 5a), and by about 4% between 10% and 50% light availability levels for Douglas fir (Figure 5b). This impact was not statistically different for Norway spruce and European beech (Table S3).

Similar to the biomass allocation to aboveground, for 10% light availability and a similar competitor, the median root mass fraction (RMF) of European beech was approximately 24% higher than the RMF of the two conifers, which allocated only about a fifth of their biomass to roots (Table S4).

**Figure 6:**
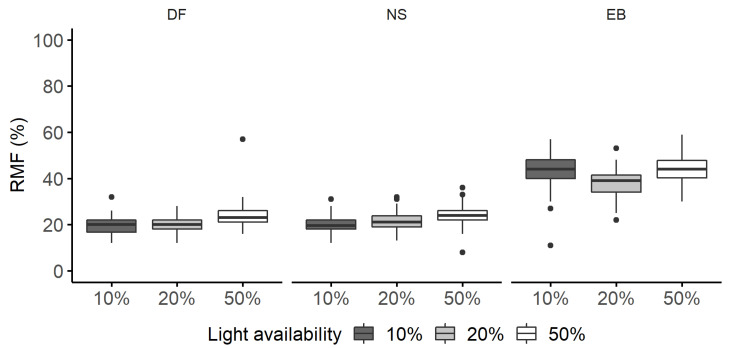
Root mass fraction (RMF, %) of Douglas fir (DF), Norway spruce (NS), and European beech (EB) saplings grown under 10%, 20%, and 50% light availability levels. Boxplots are based on raw data and graphically depict the RMF dataset. Boxplots are not an outcome of the quantile regression models.

**Figure 7:**
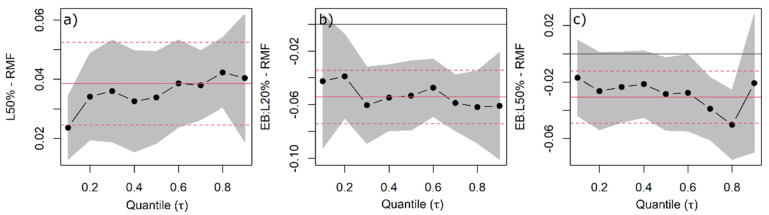
Quantile regression (QR) was used to estimate root mass fraction (RMF) changes as a function of species, light availability, and competitors’ functional similarity/dissimilarity. QR coefficients of 50% light availability common to all species (**a**), and European beech under 20% (**b**) and 50% light availability levels (**c**) are shown with the black dots indicating the estimated coefficient for each quantile and the line indicating the trend with increasing quantiles. The grey band around the dotted line represents the 95% confidence interval for the coefficients. The solid red line is the ordinary least squares (OLS) regression coefficient, and the red dashed lines represent the 95% confidence interval based on OLS.

Douglas fir and Norway spruce increased their RMF with increasing light availability (Figure 6). For both species, the effect of light was only significant with 50% light availability, with saplings allocating 3.8% more biomass to the median belowground when the light availability was high (Figure 7a).

European beech did not react to increasing light availability by gradually increasing RMF (Figure 7b,c). The median RMF increased only by 0.5% between 10% and 50% light availability levels, and the change was negligible. The saplings had the lowest allocation to RMF (39%) under a 20% light availability level (Figure 6 and Figure 7b).

As similar for LMF, the effects of sapling size on biomass allocation to woody AMF and RMF were statistically significant; however, at the median, a 1 cm increase in height was associated with only a 0.1% increase in AMF (Table S6). The height and diameter effect sizes were equally low for RMF (Table S7). Due to the small associated effect size, the height and diameter variables were considered not meaningful in explaining biomass allocation to the aboveground and root compartments, excluded from the model, and not discussed further.

### 2.3. Competitor’s Effect on Biomass Allocation

The competitor’s identity influenced biomass allocation to leaves/needles but not the woody aboveground compartments (Tables S2 and S3) in a quantile regression setting.

**Figure 8:**
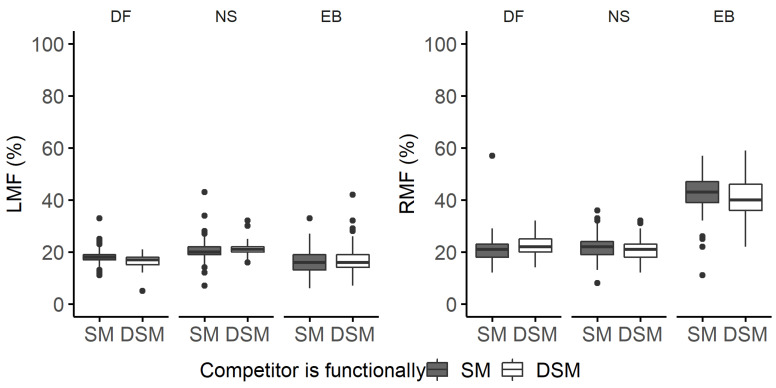
Leaf mass fraction (LMF, %) and root mass fraction (RMF, %) of Douglas fir (DF), Norway spruce (NS), and European beech (EB) saplings competing with either functionally similar (SM) or dissimilar (DSM) species. Boxplots are based on raw data and graphically depict the LMF and RMF datasets. Boxplots are not an outcome of the quantile regression models.

**Figure 9:**
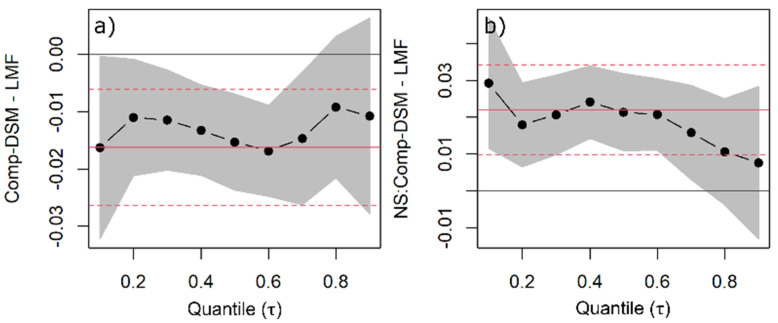
Quantile regression (QR) was used to estimate changes in leaf mass fraction (LMF) as a function of species, light availability, and competitors’ functional similarity/dissimilarity. QR coefficients of functionally dissimilar (Comp-DSM) competitors common to all species (**a**) and NS competing with functionally dissimilar species (**b**) are shown with the black dots indicating the estimated coefficient for each quantile and the line indicating the trend with increasing quantiles. The grey band around the dotted line represents the 95% confidence interval for the coefficients. The solid red line is the ordinary least squares (OLS) regression coefficient, and the red dashed lines represent the 95% confidence interval based on OLS.

The response to the competitor’s identity (functionally similar vs. functionally dissimilar) varied between species. Douglas fir allocated approximately 2% less biomass to needles if the competitor was a functionally dissimilar species (Figure 8 and Figure 9a). Norway spruce allocated slightly more biomass to needles if the competitor was functionally dissimilar, although this impact becomes approximately 0% for higher quantiles and is negligible in absolute terms (Figure 9b). For European beech, the impact of the competitor’s identity was negligible for all quantiles (Table S2).

We only observed a significant competitor’s effect on biomass allocation to roots for European beech (Table S4). The species allocated 3.3% less biomass to the median RMF, and this effect became stronger towards lower quantiles (Table S4).

The competitor’s identity affected the leaf morphology of European beech, namely the 0.5 and 0.7 quantiles, with saplings having an approximately 7 cm^2^ g^−1^ higher median SLA when the competitor was functionally dissimilar (Table S1) and tended to increase for higher quantiles.

## 3. Discussion

### 3.1. Specific Leaf Area and Leaf Mass Fraction

Considering ontogenetically similar plants, we found that all species reacted to limited light availability by increasing their SLA. Additionally, we could see pronounced differences in SLA between phyletically and functionally dissimilar conifer species—Douglas fir and Norway spruce—and the broadleaf species—European beech.

The differences in SLA between the two conifers and European beech were expected since SLA is negatively correlated with long leaf life-spans and, therefore, is generally lower for evergreens than deciduous species [44,45]. Increasing SLA with decreasing light availability is a typical and highly plastic response. It has been repeatedly reported as one of the first adaptation mechanisms to set in when the light availability decreases [25,30,46,47,48,49]. Changing SLA is a lower-cost shade-tolerance response than changing plant organs with woody tissue [50]. Moreover, a higher SLA allows plants to capture light more efficiently and, thus, increase their fitness [11]. Plasticity in SLA is generally higher than in LMF, allowing for more variability in the foliar functional traits [5].

Changes in LMF were not as pronounced as in SLA, both across light levels and between species, even though the conifers had, on average, a slightly higher LMF than European beech. This finding contradicts other studies that have consistently reported increased LMF with decreasing light availability [12,19,30]. Nevertheless, others still did not find the differences in biomass allocation to leaves or needles in high and low light availability [51]. This might result from the different adaptation mechanisms employed by the saplings of different species and present a mechanism where low biomass allocation to leaves or needles is compensated by high SLA [52].

European beech had the most substantial increase in SLA with decreasing light and the lowest LMF, which could confirm the compensation mechanism and agrees with the previously reported high phenotypic plasticity of morphological variables of European beech [8,18,48].

Conclusively, we could support our hypothesis that the LMF and SLA would increase with decreasing light availability only for the SLA and not for LMF. Even though there was an increase in the LMF of European beech and Douglas fir, it was minor overall, and the LMF was the highest under 20% light availability.

### 3.2. Woody Aboveground and Root Mass Fractions

Our results suggest that in terms of woody biomass allocation, biomass allocation patterns were primarily determined by the species’ functional group/phyletic affiliation and only secondarily by the light availability. On average, conifer species allocated 59.5% of their biomass to woody AMF, and European beech allocated 41.3% to woody AMF. At the same time, the RMF of European beech was, on average, nearly twice as high (42.5%) as the RMF of the two conifers (21.4%, not different between species).

Even though interspecific differences were pronounced, light availability could explain the intraspecific variation in biomass allocation, i.e., all species decreased their woody AMF and increased RMF between 10% and 50% light availability levels. The changes measured up to 4% for Douglas fir and Norway spruce, and 3% for European beech (only for woody AMF). Therefore, we could support our hypothesis that woody AMF increases (and RMF decreases, accordingly) with decreasing light availability, except for European beech, which did not change the RMF along the light gradient.

Root overgrowth is a common problem reported in pot experiments [6,53,54,55,56]. During the destructive harvest of the saplings, we noticed that under 50% light availability level, the roots had overtaken the growing medium, which could have impacted biomass allocation to roots. However, we could still see a positive effect of increased light availability on biomass allocation to roots and could only guess that if saplings had more growing space, then we might have seen more considerable differences between 10% and 50% light availability levels.

Whether trees allocate their biomass by optimizing resource uptake or according to size-dependent patterns has been discussed through optimal [12] and allocational partitioning theories [15]. Both theories have gained scientific support, whereas only a few studies have considered both theories when analyzing tree growth [6,15,57,58].

From our findings, we could see increased biomass allocation to (woody) aboveground biomass with decreasing light availability. This is in line with several studies that found increased biomass allocation to aboveground with decreasing light availability and confirms the optimal partitioning theory [48,59]. The findings suggest that the optimal partitioning theory can be used to predict plant responses to the presence of various environmental stressors [6].

Allocational plasticity can be affected by ontogenic drift, plant size or, to an extent, be genetically predetermined [57]. Other studies on biomass allocation in plants have repeatedly emphasized the importance of including size-related variables when analyzing whole-tree allocational patterns [16,58,60,61]. In our study with ontogenically similar saplings, the size-related variables, i.e., height and diameter, included in the models were statistically significant. However, the associated effect size was relatively small, i.e., the strength of the association was not meaningful on a practical level [62,63]. For example, a one-centimeter increase in height was associated with a change in biomass of only 0.1%. Therefore, we did not consider size as a significant predictor for biomass allocation in the early tree development stages. Suppose we follow the theory that optimal partitioning is size-independent and allocational—size-dependent [58]—then we get additional evidence supporting the optimal partitioning theory and balanced growth hypothesis. Conclusively, we could support our hypothesis that biomass allocation patterns vary between coniferous Norway spruce and Douglas fir saplings and broadleaved European beech saplings, but not that they depend on sapling size (allocational partitioning theory).

Changes in allocation imply tradeoffs as the growth and resource accumulation to one organ is done at the expense of an increase in other organs and, therefore, based on the extent of these changes, can reveal different growth strategies [58]. We can argue that the inherited phyletic differences between the two conifer species and European beech will always be present since conifers and broadleaves have different early growth patterns [28,64]. Additionally, the higher shade tolerance of European beech could add to a generally lower biomass allocation to aboveground, and less pronounced changes in biomass allocation patterns along the light gradient, as the species employ different strategies to adapt to limited light than changing the biomass [19,49,65]. Growth patterns and differences in biomass allocation patterns could also be affected by polycyclism, i.e., the plant’s ability to produce several flushes in one growing season [66]. Polycyclic growth has been previously observed for all studied species [67,68,69]. Plants with polycyclic growth tend to have a higher branching rate, which increases the aboveground biomass proportion, and its rate is higher early in ontogeny [70]. Further studies about the potential effect of polycyclism on biomass allocation are needed since it is an understudied mechanism, especially in connection with biomass allocation.

### 3.3. Competitor’s Effect on Biomass Allocation

To increase the model’s comprehensibility and look beyond the direct impact of limited light availability on individual plant traits, we also included the effect of a competitor to assess the plant’s competitive response in an environment where the already limited resources must be shared. Similar interactions in a natural environment would impact the community structure [71] and, therefore, should not be overlooked when analyzing the growth response of saplings.

Since the local light environment can be affected by the physical presence of neighbors and, therefore, impact growth [72], we expected a higher allocation to aboveground as a strategy for increasing the light interception, followed by a higher allocation to leaves/needles due to the rapid use of the captured resources [52]. The competitor’s identity or functional dissimilarity (based on phyletic affiliation) affected biomass allocation to leaves and needles but not woody aboveground mass fractions. Having a functionally dissimilar competitor resulted in higher LMF of Douglas fir (+2%) and slightly lower LMF of European beech and Norway spruce. The effect, however, was arguably minor and should be interpreted with caution. Such a finding was not surprising for lower light availability levels where sapling’s aboveground compartments rarely interacted. However, we were hoping to see an effect under the 50% light availability level due to the encroachment of neighboring trees. Nevertheless, the interaction term with light did not change the outcome and was excluded from the model.

The higher LMF of Douglas fir competing with functionally dissimilar species—European beech—could potentially indicate the improved growing ability of Douglas fir due to the complementarity of the two species. A similar positive mixture effect has been previously reported in the literature, including the overyielding analysis of this pot experiment, and found a higher biomass production in the mixed Douglas fir and European beech pots than in monospecific pots of either species [73].

Against this background, the results support our hypothesis that individuals competing with functionally dissimilar species would allocate more biomass to leaf, woody aboveground, and root biomass fractions than individuals competing with functionally similar species, due to the decreased competition pressure (for the access to above- and below-ground resources), for only Douglas fir and the LMF. In contrast, the effect of a functionally dissimilar competitor on the RMF of European beech was negative. We assume that the trends observed in the competitor effect analysis might lead to more substantial effects as the saplings age. Moreover, biomass allocation to woody organs might be influenced by the nursery legacy effect and could still determine the general allocation patterns two years after growing under controlled conditions [74].

## 4. Materials and Methods

### 4.1. Study Site and Experimental Setup

We conducted a controlled pot experiment at the University of Göttingen (51°33′21.6″ N 9°57′11.8″ E). The study site was located 174 m above sea level. The climate is classified as temperate and oceanic [75]. The average annual temperature is 9.2 °C, and the precipitation reaches 650 mm per annum, approximately half of which falls between May to September (www.dwd.de, accessed on 16 March 2021).

We filled 65l pots (H = 33 cm; D = 56 cm) with a 5 cm coarse gravel layer, then a layer of thin fleece and nutrient-poor sand. The coarse gravel layer provided better drainage, while the fleece layer stopped sand from leaching out. We chose sand as the potting medium to facilitate the planned destructive harvest. The filled pots were then arranged on wooden laths for additional gravity drainage. All pots received the same water treatment through an automatic drip irrigation system and the precipitation that reached the seedlings. The poor growing medium was supplied with nutrients using controlled-release fertilizer Osmocote Exact Hi.End with 12–14 months longevity (ICL SF, Nordhorn, Germany).

**Figure 10:**
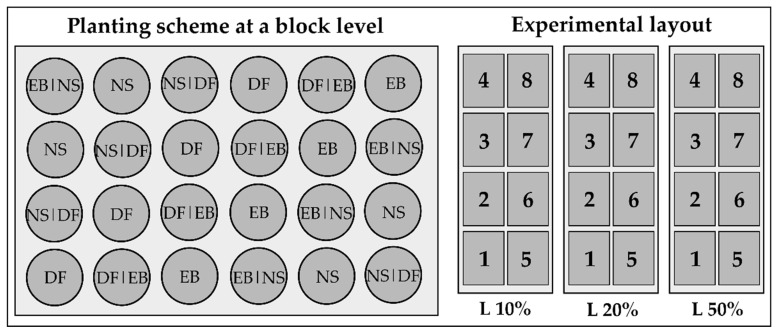
A block-wise planting scheme and the layout of the pot experiment. Each block consisted of 24 pots arranged in a mixed and systematic way based on the planting combination. There were eight blocks under each light treatment. DF—monospecific Douglas fir pot, DF|EB—mixed Douglas fir and European beech pot, EB—monospecific European beech pot, EB|NS—mixed European beech and Norway spruce pot, NS—monospecific Norway spruce pot, NS|DF—mixed Norway spruce and Douglas fir pot. Numbers 1–8 indicate the blocks under each light treatment. L10%—10% light availability level, L20%—20% light availability level, L50%—50% light availability level.

European beech (EB), Norway spruce (NS), and Douglas fir (DF) seedlings were planted in monospecific (EB, NS, DF) and mixed (DF + EB, EB + NS, NS + DF) pots with 32 replicates per treatment (light × composition type). There were four seedlings per pot. The seedlings were planted in a square-shaped layout in mixed pots, and seedlings of the same species were planted diagonally. We planted 576 pots arranged in eight 24-pot blocks per light treatment—3 light availability levels × 6 planting combinations × 32 replicates (Figure 10).

The seedlings came from a local nursery (Baumschule Willenbocke GmbH, Walsrode, Germany). Before planting, European beech seedlings were 1 year old (ranging from 22–56 cm), whereas Norway spruce (ranging from 10–48 cm) and Douglas fir (ranging from 11–30 cm) seedlings were 2 years old. The seedlings were not undercut or transplanted, thus ensuring that previous treatments at the nursery had an as minimal legacy effect on plant organs (particularly roots) as possible.

The used light availability levels were 10%, 20%, and 50%. Such levels were chosen to mimic the light availability in gaps of various sizes [76]. The lower light availability levels (10% and 20%) aimed to expose saplings to low enough light availability to see shifts in biomass allocation without causing high mortality. The intermediately high light availability level of 50% was chosen to represent good growing conditions for more shade-tolerant and moderately shade-tolerant species, thus diversifying between the effects of the light environments between the species [17,77,78]. Light availability levels were determined as the proportion of irradiance under the shading nets to irradiance under open field conditions. As we were only interested in the proportion of irradiance reaching plants under the shading nets compared to ambient conditions, the irradiance was measured in a single 2 h campaign. The measurements were taken every 5 min, moving on a transect across the experimental blocks. The irradiance was simultaneously measured on an open field. Irradiance was measured with an LI-COR QUANTUM^®^ sensor (LI-COR Biosciences, Lincoln, NE, USA).

### 4.2. Sampling Procedure

We grew the saplings for two vegetation periods (April 2018–October 2019) and harvested them at the end of the second vegetation period in 2019. Only pots containing four live and vital saplings were harvested to avoid the effect of increased growing space or reduced competition on the biomass allocation. There was a substantial die-off of seedlings due to planting shock (mainly for Douglas fir) and a loss of vitality due to extensive infestations of woolen aphid (*Phyllaphis fagi* L.) and brown spruce shoot aphid (*Cinara pilicornis* Hartig). The mortality rate of Douglas fir saplings was 38%, whereas the mortality of European beech and Norway spruce was under 1%. The pots with saplings that were alive but did not have any increments, or pots that had live saplings, but the leaves were too damaged due to aphid infestation (mainly in the case of European beech), were also not selected for harvest. An additional 126 pots were harvested for other experimental purposes. This resulted in the final harvest of 202 pots from the 576 pots initially planted.

The harvested saplings were washed and separated by their compartments—leaves/needles, branches, stems, and roots. The samples were dried at 70 °C for 72 h until a constant weight was reached. The samples were then weighed using a standard laboratory scale Sartorius MC1 LC1200S^®^ with an accuracy of ±0.003 g (Sartorius AG, Göttingen, Germany).

The leaves of European beech saplings were sampled for a leaf area estimation before the sapling harvest. At the same time, the needles of Norway spruce and Douglas fir saplings were sampled in the lab after the sapling harvest. Representative samples of 20 leaves or 40 needles per sapling were collected and scanned with an office-type scanner (CANON LIDE 300). The dataset is openly available in the PANGAEA data repository [79].

### 4.3. Data Processing

The leaf area was determined by processing the images of scanned samples with WinFolia 2004a (Régent Instruments Inc., Quebec, Canada) software. The sapling SLA was calculated as the leaf area divided by the oven-dry leaf mass (Equation (1)).

Biomass fractions were calculated as the proportion of a biomass fraction to the total woody plant biomass. Woody AMF was calculated as the sum of branch and stem mass fractions (Equation (2)). The following formulas were used to calculate the SLA and biomass fractions:

(1) SLA = specific leaf area (cm^2^ g^−1^) = LA/LM_d_

(2) tC_MF = tree compartment mass fraction (g g^−1^) = BM_tC/BM_tot

where LA = leaf area (cm^2^), LM_d_ = oven-dry leaf mass (g), BM_tC = oven-dry biomass of a tree compartment (g), and BM_tot = oven-dry total plant biomass (g).

Light availability, species identity, and competitor’s functional similarity were the main explanatory variables used in the analysis. For mixed planting combinations, the competitor’s identity was determined as the species of the two saplings closest to the target sapling, i.e., the other species. If the competitor was the same species or another conifer, it was functionally similar. However, it was considered functionally dissimilar if the competitor was a different species or species group, i.e., broadleaf (European beech) and conifer (Douglas fir or Norway spruce) mixtures. The functional similarity or dissimilarity was based on phyletic affiliation, i.e., conifers and broadleaves. The model’s baseline group represents Douglas fir, under 10% light availability, and with functionally similar competitors.

The number of observations of SLA, LMF, woody AMF, and RMF varied due to removed measurement errors, especially for needle samples when estimating SLA, and a lower number of vital Douglas fir saplings (Table S8).

### 4.4. Statistical Analysis

Quantile regression (QR) is used in the statistical analysis of SLA, LMF, woody AMF, and root mass fraction (RMF) [80,81]. Typically, ordinary least squares (OLS) are utilized in the estimation of regression models. However, while OLS (only) models the mean of the response distribution (SLA, LMF, woody AMF, or RMF) in the dependence of certain covariates (e.g., light availability), QR directly models the quantiles of the response distribution [82,83]. Contrary to OLS, QR does not resort to specific parametric distribution families, i.e., has no distributional assumptions, and it can be used when the assumptions of OLS are not met. Thus, QR is more flexible than OLS: it can be used without the extensive model checks that OLS requires and is able to identify dependence beyond the mean. Moreover, in our case, it can also be applied to both bounded (e.g., RMF) and unbounded response domains (e.g., SLA).

The regression coefficients in QR can be interpreted similarly to OLS’, but instead refer to a given quantile (τ) of the response distribution and not to its mean. Given this, when considering a logarithmic transformation of the response, the (approximate) interpretation of the associated change in the quantile τ of the response is:

$$\%\Delta response\;\approx \;\left(100\;\cdot \;coefficient\right)\Delta \mathrm{covariate}$$

where Δresponse and Δcovariate  represent the change in response and covariate, respectively [84]. Concretely, this means that a one-unit change in the covariate is associated with a (100 · coefficient)% change in the considered quantile of the response.

Concretely, herein, we estimate five quantiles: τ = 0.1, 0.3, 0.5, 0.7, 0.9. In what follows, we will interpret the results at the median (τ = 0.5) while pointing out situations in which coefficient estimates change significantly between quantiles. We have provided an example for the interpretation of QR coefficients in the Supplementary Materials (Supplement S9). We consider equal-tailed 95% confidence intervals, i.e., α = 0.05. Additionally, throughout the analysis, we also provide OLS results as a means of comparison.

All statistical analyses and tests were performed in the statistical computing software environment R, version 4.0.2 [85]. Quantile regression was performed using the R-package “quantreg” [86].

## 5. Conclusions

Biomass allocation patterns and leaf morphology were primarily determined by a species’ functional group (conifers vs. broadleaves) and only secondly by light availability. Even though the effect of light on biomass allocation was not very pronounced and amounted to changes of a few percent, it could explain some of the residual intraspecific variation.

We found pronounced differences between SLA, woody AMF, and RMF of the two conifer species and European beech, with European beech having higher SLA and RMF and the conifers having higher woody AMF across the light levels.

Of all the variables, SLA showed the strongest reaction and increased with decreasing light availability for all species, whereas a more minor effect of light availability was observed for woody organs. LMF increased with decreasing light availability for only Douglas fir and European beech, but was the highest at 20% light availability. The plasticity in SLA might have compensated for the minor changes in LMF.

The effect size associated with the size-related height and diameter variables was small and considered not meaningful in explaining biomass allocation on a practical level. The competitor’s effect on biomass allocation was minimal. Douglas fir allocated more biomass to needles when the competitor was functionally dissimilar than when the competitor was functionally similar. In contrast, the trend was opposite (yet not significant) for European beech and Norway spruce. The competitor’s effect on biomass allocation to roots was significant only for European beech, with species allocating less biomass to roots when competing with functionally dissimilar species. The facilitating effect of growing functionally dissimilar species together was not reflected through changing biomass allocation patterns or detected in the early tree development stages.

The overall minor changes in biomass allocation could have resulted from the nursery legacy effect, since the light conditions under which seedlings were grown in the nursery can affect the growth and biomass allocation of young trees for some time after planting. As a result, pronounced changes in biomass allocation might only be observable in later development stages. The potential delayed effects to limited-resource environments should be considered when assessing early regeneration success.

## Data Availability

The datasets generated during the current study are available from PANGAEA^®^ Data Publisher (https://doi.pangaea.de/10.1594/PANGAEA.933150). The code is available from the corresponding author on reasonable request.

## Supplementary Materials

The following are available online at https://www.mdpi.com/article/10.3390/plants11030305/s1, Table S1: Regression coefficients for different quantiles (τ) in the quantile regression and ordinary least squares (OLS) models for specific leaf area (SLA), Table S2: Regression coefficients for different quantiles (τ) in the quantile regression and ordinary least squares (OLS) models for leaf mass fraction (LMF), Table S3: Regression coefficients for different quantiles (τ) in the quantile regression and ordinary least squares (OLS) models for woody aboveground mass fraction (AMF), Table S4: Regression coefficients for different quantiles (τ) in the quantile regression and ordinary least squares (OLS) models for root mass fraction (RMF). Table S5: Regression coefficients for different quantiles (τ) in the quantile regression and ordinary least squares (OLS) models for leaf mass fraction (LMF) including height and diameter variables. Table S6: Regression coefficients for different quantiles (τ) in the quantile regression and ordinary least squares (OLS) models for woody aboveground mass fraction (AMF) including height and diameter variables. Table S7: Regression coefficients for different quantiles (τ) in the quantile regression and ordinary least squares (OLS) models for root mass fraction (RMF) including height and diameter variables. Table S8. Number or replicates per species and light level used to analyze leaf morphology and biomass allocation. Supplement S9. The interpretation of quantile regression coefficients based on Tables S1–S7.
