# Supplementary material for: Biomass Allocation and Leaf Morphology of Saplings Grown under Various Conditions of Light Availability and Competition Types
Source: Plants (Basel). 2022 Jan 24;11(3):305. doi: 10.3390/plants11030305 (PMC8839049; doi:10.3390/plants11030305)
Supplement: Supplementary file 1 [file plants-11-00305-s001.zip › plants-1552552-supplementary.pdf]

Table S1: Regression coefficients for different quantiles (τ) in the quantile regression and ordinary least squares (OLS) models for Specific Leaf Area (SLA). Standard errors are shown in brackets. Asterisks indicate the p-value (p) group: * p <0.1, ** p <0.05, *** p <0.01.

	Dependent variable: Specific Leaf Area (SLA)					
	**OLS**	**τ = 0.1**	**τ = 0.3**	**τ = 0.5**	**τ = 0.7**	**τ = 0.9**
Intercept	116.830***	96.326***	112.337***	112.337***	127.338***	127.338***
	(12.321)	(12.748)	(14.876)	(15.173)	(10.532)	(18.441)
European beech	127.917***	95.798***	122.547***	135.596***	134.210***	163.524***
	(12.643)	(16.719)	(18.013)	(15.623)	(13.145)	(19.232)
Norway spruce	-14.331	−5.860	−14.321	−10.494	−17.876	−12.638
	(15.697)	(12.957)	(15.630)	(15.525)	(12.163)	(18.804)
Light 20%	-22.976*	−12.331	−24.409	−20.603	−28.336***	−25.380
	(13.060)	(12.956)	(15.103)	(15.255)	(10.649)	(19.690)
Light 50%	-37.153***	−25.885**	−38.770***	−35.026**	−42.431***	−37.802**
	(12.736)	(12.794)	(14.866)	(15.077)	(10.923)	(18.655)
Competitor – dissimilar	-3.255	−4.263*	−1.750	−2.758	−5.570	0.725
	(7.242)	(2.495)	(2.924)	(3.518)	(4.744)	(7.248)
European beech: Light 20%	-68.133***	−47.429***	−60.241***	−72.227***	−72.877***	−96.369***
	(13.565)	(17.727)	(18.629)	(15.632)	(13.044)	(20.686)
Norway spruce: Light 20%	16.841	9.611	18.075	15.475	19.196	16.323
	(16.594)	(13.275)	(15.831)	(15.524)	(12.302)	(20.086)
European beech: Light 50%	-68.734***	−47.251***	−67.967***	−78.648***	−71.702***	−98.688***
	(13.267)	(16.882)	(18.122)	(15.572)	(13.043)	(19.997)
Norway spruce: Light 50%	19.840	10.300	21.828	18.260	21.970*	18.629
	(16.122)	(13.089)	(15.658)	(15.394)	(12.372)	(19.010)
European beech: Competitor – dissimilar	10.166	7.816	3.816	10.241**	13.224**	9.132
	(7.863)	(6.329)	(5.309)	(4.419)	(5.725)	(8.361)
Norway spruce: Competitor – dissimilar	1.011	5.559*	0.709	−0.943	1.223	−4.234
	(9.065)	(2.914)	(3.262)	(3.996)	(5.551)	(7.978)
Observations	431	431	431	431	431	431

Table S2: Regression coefficients for different quantiles (τ) in the quantile regression and ordinary least squares (OLS) models for Leaf Mass Fraction (LMF). Standard errors are shown in brackets. Asterisks indicate the p-value (p) group: * p <0.1, ** p <0.05, *** p <0.01.

	Dependent variable: Leaf Mass Fraction (LMF)					
	**OLS**	**τ = 0.1**	**τ = 0.3**	**τ = 0.5**	**τ = 0.7**	**τ = 0.9**
Intercept	0.178*** (0.004)	0.143*** (0.007)	0.166*** (0.005)	0.177*** (0.004)	0.190*** (0.003)	0.211*** (0.008)
European beech	-0.040*** (0.006)	-0.054*** (0.009)	-0.046*** (0.006)	-0.041*** (0.007)	-0.036*** (0.006)	-0.023* (0.013)
Norway spruce	0.035*** (0.005)	0.028*** (0.010)	0.029*** (0.006)	0.036*** (0.006)	0.037*** (0.004)	0.041*** (0.010)
Light 20%	0.019*** (0.007)	0.020** (0.010)	0.019** (0.008)	0.017*** (0.007)	0.013* (0.007)	0.018 (0.013)
Light 50%	0.001 (0.006)	0.017** (0.008)	0.002 (0.006)	0.001 (0.005)	−0.006 (0.004)	−0.010 (0.010)
Competitor – dissimilar	-0.016*** (0.006)	-0.016 (0.012)	−0.011** (0.005)	-0.015*** (0.005)	−0.015** (0.007)	-0.011 (0.010)
European beech: Light 20%	0.047*** (0.008)	0.034*** (0.012)	0.032** (0.013)	0.047*** (0.011)	0.056*** (0.010)	0.063*** (0.018)
Norway spruce: Light 20%	-0.026*** (0.008)	-0.014 (0.012)	-0.020** (0.008)	-0.026*** (0.008)	0.019** (0.008)	-0.036** (0.014)
European beech: Light 50%	0.022*** (0.008)	0.014 (0.011)	0.019* (0.010)	0.024*** (0.008)	0.029*** (0.008)	0.026 (0.016)
Norway spruce: Light 50%	-0.017** (0.007)	-0.018 (0.011)	-0.012* (0.007)	-0.017*** (0.007)	-0.013** (0.006)	-0.008 (0.012)
European beech: Competitor – dissimilar	0.018** (0.007)	0.019 (0.013)	0.012 (0.009)	0.018** (0.008)	0.016 (0.010)	0.005 (0.014)
Norway spruce: Competitor – dissimilar	0.022*** (0.007)	0.029** (0.013)	0.021*** (0.007)	0.021*** (0.006)	0.016* (0.009)	0.008 (0.013)
Observations	773	773	773	773	773	773

Table S3: Regression coefficients for different quantiles (τ) in the quantile regression and ordinary least squares (OLS) models for woody Aboveground Mass Fraction (AMF). Standard errors are shown in brackets. Asterisks indicate the p-value (p) group: * p <0.1, ** p <0.05, *** p <0.01.

	Dependent variable: Woody Aboveground Mass Fraction (AMF)					
	**OLS**	**τ = 0.1**	**τ = 0.3**	**τ = 0.5**	**τ = 0.7**	**τ = 0.9**
Intercept	0.630*** (0.006)	0.605*** (0.015)	0.621*** (0.005)	0.631*** (0.006)	0.647*** (0.005)	0.661*** (0.004)
European beech	-0.205*** (0.007)	-0.242*** (0.015)	-0.223*** (0.010)	-0.202*** (0.011)	-0.197*** (0.010)	-0.174*** (0.012)
Norway spruce	-0.041*** (0.007)	-0.048*** (0.016)	-0.041*** (0.005)	-0.040*** (0.006)	-0.044*** (0.006)	-0.037*** (0.006)
Light 20%	-0.018** (0.008)	-0.030 (0.022)	-0.026*** (0.007)	-0.020** (0.009)	-0.016** (0.006)	-0.009 (0.011)
Light 50%	-0.035*** (0.006)	-0.050*** (0.008)	-0.043*** (0.006)	-0.035*** (0.005)	−0.033*** (0.004)	−0.027*** (0.010)
Competitor – dissimilar	-0.004 (0.008)	-0.035 (0.023)	−0.004 (0.010)	-0.003 (0.007)	−0.001 (0.007)	-0.006 (0.012)
European beech: Light 20%	0.009 (0.011)	0.008 (0.023)	0.011 (0.012)	0.008 (0.014)	0.010 (0.014)	0.010 (0.021)
Norway spruce: Light 20%	0.008 (0.010)	0.018 (0.024)	0.013* (0.008)	0.005 (0.010)	0.005 (0.008)	-0.001 (0.014)
European beech: Light 50%	0.010 (0.010)	0.034 (0.022)	0.009 (0.012)	-0.001 (0.013)	0.014 (0.020)	0.013 (0.019)
Norway spruce: Light 50%	0.008 (0.009)	0.011 (0.020)	0.013 (0.009)	0.004 (0.008)	0.005 (0.009)	0.004 (0.013)
European beech: Competitor – dissimilar	0.017* (0.010)	0.041 (0.025)	0.019 (0.014)	0.004 (0.012)	0.021 (0.016)	0.031 (0.020)
Norway spruce: Competitor – dissimilar	0.008 (0.010)	0.027 (0.025)	0.009 (0.010)	0.002 (0.009)	0.004 (0.007)	0.0001 (0.015)
Observations	761	761	761	761	761	761

Table S4: Regression coefficients for different quantiles (τ) in the quantile regression and ordinary least squares (OLS) models for Root Mass Fraction (RMF). Standard errors are shown in brackets. Asterisks indicate the p-value (p) group: * p <0.1, ** p <0.05, *** p <0.01.

	Dependent variable: Root Mass Fraction (RMF)					
	**OLS**	**τ = 0.1**	**τ = 0.3**	**τ = 0.5**	**τ = 0.7**	**τ = 0.9**
Intercept	0.194***<br>(0.006)	0.155***<br>(0.006)	0.170***<br>(0.008)	0.198***<br>(0.006)	0.214***<br>(0.004)	0.233***<br>(0.010)
European beech	0.244***<br>(0.009)	0.221***<br>(0.012)	0.242***<br>(0.011)	0.243***<br>(0.011)	0.262***<br>(0.009)	0.287***<br>(0.015)
Norway spruce	0.005<br>(0.008)	-0.004<br>(0.009)	0.010<br>(0.009)	-0.0002<br>(0.007)	0.005<br>(0.006)	0.007<br>(0.013)
Light 20%	-0.0004<br>(0.010)	-0.014<br>(0.014)	0.012<br>(0.012)	-0.001<br>(0.008)	-0.003<br>(0.007)	-0.001<br>(0.016)
Light 50%	0.039***<br>(0.008)	0.024***<br>(0.009)	0.036***<br>(0.010)	0.034***<br>(0.010)	0.038***<br>(0.007)	0.040***<br>(0.013)
Competitor – dissimilar	0.015*<br>(0.009)	0.016<br>(0.011)	0.020*<br>(0.010)	0.014*<br>(0.008)	0.010<br>(0.011)	0.030<br>(0.020)
European beech: Light 20%	-0.054***<br>(0.012)	-0.043<br>(0.030)	-0.060***<br>(0.016)	-0.053***<br>(0.015)	-0.059***<br>(0.012)	-0.061***<br>(0.021)
Norway spruce: Light 20%	0.017<br>(0.012)	0.033**<br>(0.016)	0.009<br>(0.014)	0.019**<br>(0.010)	0.014<br>(0.010)	0.015<br>(0.018)
European beech: Light 50%	-0.031***<br>(0.010)	-0.017<br>(0.022)	-0.024<br>(0.012)	-0.029*<br>(0.013)	-0.039***<br>(0.020)	-0.021<br>(0.019)
Norway spruce: Light 50%	0.004<br>(0.011)	0.026*<br>(0.014)	0.004<br>(0.012)	0.007<br>(0.011)	0.003<br>(0.010)	0.017<br>(0.018)
European beech: Competitor – dissimilar	-0.030***<br>(0.011)	-0.052**<br>(0.021)	-0.039***<br>(0.015)	-0.033***<br>(0.012)	-0.022*<br>(0.013)	-0.038<br>(0.024)
Norway spruce: Competitor – dissimilar	-0.022**<br>(0.011)	-0.023<br>(0.015)	-0.023*<br>(0.012)	-0.018*<br>(0.010)	-0.017<br>(0.012)	-0.038*<br>(0.022)
Observations	773	773	773	773	773	773

Table S5: Regression coefficients for different quantiles (τ) in the quantile regression and ordinary least squares (OLS) models for Leaf Mass Fraction (LMF) including height and diameter variables. Standard errors are shown in brackets. Asterisks indicate the p-value (p) group: * p <0.1, ** p <0.05, *** p <0.01.

	Dependent variable: Leaf Mass Fraction (LMF)					
	**OLS**	**τ = 0.1**	**τ = 0.3**	**τ = 0.5**	**τ = 0.7**	**τ = 0.9**
Intercept	0.186*** (0.006)	0.142*** (0.008)	0.172*** (0.007)	0.190*** (0.006)	0.201*** (0.006)	0.229*** (0.009)
European beech	-0.040*** (0.006)	-0.053*** (0.009)	-0.046*** (0.007)	-0.042*** (0.007)	-0.034*** (0.006)	-0.027** (0.011)
Norway spruce	0.032*** (0.006)	0.028*** (0.010)	0.026*** (0.006)	0.029*** (0.005)	0.034*** (0.005)	0.032*** (0.009)
Light 20%	0.023*** (0.007)	0.020** (0.009)	0.022** (0.008)	0.022*** (0.006)	0.020*** (0.007)	0.021* (0.011)
Light 50%	0.009 (0.008)	0.013 (0.011)	0.008 (0.009)	0.002 (0.005)	0.005 (0.007)	0.004 (0.013)
Competitor – dissimilar	-0.015** (0.006)	-0.016 (0.012)	−0.015*** (0.005)	-0.012** (0.005)	−0.011* (0.006)	-0.012 (0.009)
**Height (cm)**	**-0.0002* (0.0001)**	**-0.0001 (0.0001)**	**-0.0002* (0.0001)**	**-0.0003*** (0.0001)**	**-0.0002** (0.0001)**	**-0.0004** (0.0002)**
**Diameter (mm)**	**0.0001 (0.001)**	**0.001 (0.001)**	**0.001 (0.001)**	**0.001* (0.001)**	**0.00002 (0.001)**	**0.0003 (0.001)**
European beech: Light 20%	0.047*** (0.008)	0.034*** (0.012)	0.032** (0.013)	0.047*** (0.011)	0.056*** (0.010)	0.063*** (0.018)
Norway spruce: Light 20%	-0.026*** (0.008)	-0.014 (0.012)	-0.020** (0.008)	-0.026*** (0.008)	0.019** (0.008)	-0.036** (0.014)
European beech: Light 50%	0.022*** (0.008)	0.014 (0.011)	0.019* (0.010)	0.024*** (0.008)	0.029*** (0.008)	0.026 (0.016)
Norway spruce: Light 50%	-0.017** (0.007)	-0.018 (0.011)	-0.012* (0.007)	-0.017*** (0.007)	-0.013** (0.006)	-0.008 (0.012)
European beech: Competitor – dissimilar	0.018** (0.007)	0.019 (0.013)	0.012 (0.009)	0.018** (0.008)	0.016 (0.010)	0.005 (0.014)
Norway spruce: Competitor – dissimilar	0.022*** (0.007)	0.029** (0.013)	0.021*** (0.007)	0.021*** (0.006)	0.016* (0.009)	0.008 (0.013)
Observations	773	773	773	773	773	773

Table S6: Regression coefficients for different quantiles (τ) in the quantile regression and ordinary least squares (OLS) models for woody Aboveground Mass Fraction (AMF) including height and diameter variables. Standard errors are shown in brackets. Asterisks indicate the p-value (p) group: * p <0.1, ** p <0.05, *** p <0.01.

	Dependent variable: Woody Aboveground Mass Fraction (AMF)					
	**OLS**	**τ = 0.1**	**τ = 0.3**	**τ = 0.5**	**τ = 0.7**	**τ = 0.9**
Intercept	0.588***	0.553***	0.573***	0.590***	0.611***	0.634***
	(0.007)	(0.012)	(0.007)	(0.007)	(0.009)	(0.010)
European beech	-0.206***	-0.235***	-0.218***	-0.206***	-0.209***	-0.187***
	(0.007)	(0.009)	(0.007)	(0.008)	(0.009)	(0.013)
Norway spruce	-0.023***	-0.016	-0.019***	-0.021***	-0.029***	-0.026***
	(0.007)	(0.011)	(0.005)	(0.006)	(0.007)	(0.009)
Light 20%	-0.038***	-0.047***	-0.042***	-0.032***	-0.032***	-0.020*
	(0.008)	(0.011)	(0.007)	(0.011)	(0.008)	(0.011)
Light 50%	-0.064***	-0.078***	-0.069***	-0.061***	−0.054***	−0.046***
	(0.009)	(0.019)	(0.010)	(0.011)	(0.012)	(0.014)
Competitor – dissimilar	-0.008	-0.032*	−0.008	-0.009	−0.002	-0.009
	(0.007)	(0.019)	(0.006)	(0.009)	(0.007)	(0.009)
**Height (cm)**	**0.001*** **	**0.001*** **	**0.001*** **	**0.001*** **	**0.001*** **	**0.001*** **
	**(0.0001)**	**(0.0002)**	**(0.0002)**	**(0.0002)**	**(0.0002)**	**(0.0002)**
**Diameter (mm)**	**-0.003*** **	**-0.005*** **	**-0.002***	**-0.004*** **	**-0.003*** **	**-0.002**
	**(0.001)**	**(0.002)**	**(0.001)**	**(0.001)**	**(0.001)**	**(0.002)**
European beech: Light 20%	0.024**	0.037***	0.024**	0.018	0.028**	0.012
	(0.010)	(0.014)	(0.012)	(0.014)	(0.012)	(0.019)
Norway spruce: Light 20%	0.024**	0.031**	0.023***	0.015	0.021**	0.004
	(0.010)	(0.012)	(0.008)	(0.012)	(0.008)	(0.015)
European beech: Light 50%	0.023**	0.032*	0.024**	0.011	0.024	0.016
	(0.010)	(0.018)	(0.012)	(0.013)	(0.017)	(0.019)
Norway spruce: Light 50%	0.027***	0.025	0.022***	0.028***	0.022**	0.020
	(0.009)	(0.018)	(0.008)	(0.010)	(0.010)	(0.014)
European beech: Competitor – dissimilar	0.012	0.036	0.014	0.009	0.016	0.032**
	(0.009)	(0.022)	(0.009)	(0.011)	(0.011)	(0.016)
Norway spruce: Competitor – dissimilar	0.009	0.033	0.011	0.009	0.002	0.005
	(0.009)	(0.021)	(0.008)	(0.010)	(0.008)	(0.012)
Observations	761	761	761	761	761	761

Table S7: Regression coefficients for different quantiles (τ) in the quantile regression and ordinary least squares (OLS) models for Root Mass Fraction (RMF) including height and diameter variables. Standard errors are shown in brackets. Asterisks indicate the p-value (p) group: * p <0.1, ** p <0.05, *** p <0.01.

	Dependent variable: Root Mass Fraction (RMF)					
	**OLS**	**τ = 0.1**	**τ = 0.3**	**τ = 0.5**	**τ = 0.7**	**τ = 0.9**
Intercept	0.230*** (0.008)	0.179*** (0.011)	0.193*** (0.012)	0.229*** (0.010)	0.241*** (0.008)	0.275*** (0.013)
European beech	0.245*** (0.008)	0.222*** (0.013)	0.242*** (0.010)	0.237*** (0.012)	0.264*** (0.008)	0.284*** (0.015)
Norway spruce	-0.011 (0.008)	-0.012 (0.011)	0.001 (0.010)	-0.017** (0.009)	-0.008 (0.005)	-0.011 (0.011)
Light 20%	0.016* (0.010)	0.003 (0.013)	0.012 (0.013)	0.011 (0.009)	0.009 (0.009)	0.032** (0.014)
Light 50%	0.061*** (0.011)	0.036** (0.017)	0.046*** (0.012)	0.052*** (0.013)	0.056*** (0.012)	0.071*** (0.023)
Competitor – dissimilar	0.018** (0.009)	0.016 (0.011)	0.022** (0.010)	0.010 (0.009)	0.018** (0.008)	0.023 (0.015)
**Height (cm)**	**-0.001*** (0.0001)**	**-0.001*** (0.0003)**	**-0.001*** (0.0002)**	**-0.001*** (0.0002)**	**-0.001*** (0.0002)**	**-0.001*** (0.0002)**
**Diameter (mm)**	**0.003*** (0.001)**	**0.003 (0.002)**	**0.003* (0.001)**	**0.003** (0.001)**	**0.003* (0.001)**	**0.005*** (0.002)**
European beech: Light 20%	-0.067*** (0.012)	-0.064*** (0.023)	-0.054*** (0.017)	-0.056*** (0.014)	-0.064*** (0.014)	-0.088*** (0.020)
Norway spruce: Light 20%	0.003 (0.011)	0.012 (0.016)	0.007 (0.014)	0.009 (0.011)	0.010 (0.010)	-0.013 (0.015)
European beech: Light 50%	-0.041*** (0.012)	-0.025 (0.019)	-0.027 (0.016)	-0.035** (0.015)	-0.048*** (0.017)	-0.039 (0.028)
Norway spruce: Light 50%	-0.011 (0.011)	-0.014 (0.016)	-0.007 (0.012)	-0.009 (0.011)	-0.004 (0.011)	-0.010 (0.017)
European beech: Competitor – dissimilar	-0.025** (0.011)	-0.041** (0.016)	-0.040*** (0.014)	-0.022* (0.012)	-0.029** (0.014)	-0.027 (0.021)
Norway spruce: Competitor – dissimilar	-0.023** (0.010)	-0.027* (0.016)	-0.023** (0.011)	-0.010 (0.009)	-0.026*** (0.009)	-0.036** (0.017)
Observations	773	773	773	773	773	773

Table S8. Number or replicates per species and light level used to analyze leaf morphology and biomass allocation. SLA – specific leaf area, LMF – leaf mass fraction, AMF – woody aboveground mass fraction, RMF – root mass fraction.

Species	Light	SLA	LMF	AMF	RMF
Douglas fir	10%	4	60	58	60
Douglas fir	20%	27	45	45	45
Douglas fir	50%	45	72	71	72
European beech	10%	99	103	101	103
European beech	20%	80	79	77	79
European beech	50%	76	74	71	74
Norway spruce	10%	7	132	130	132
Norway spruce	20%	35	102	102	102
Norway spruce	50%	58	106	106	106

Supplement S9. The interpretation of quantile regression coefficients based on Tables S1-S7.

In the paper, we state that *"Under a 10% light availability level and similar competitor, Norway spruce allocated the most biomass to LMF (21%) compared to Douglas fir (18%) and European beech (14%)."* We use this statement as an example to explain how regression coefficients can be interpreted, given the regression tables S1-S7 provided in the supplements.

The base model represents Douglas fir, under 10% light availability and similar competitor.

Consider Table S2:

- The effect of Douglas fir on the median of LMF ($\tau = 0.5$) corresponds to $100 \cdot 0.177 \approx 18\%$, where 0.177 is the coefficient of the intercept.
- The effect of Norway spruce on the median of LMF ($\tau = 0.5$) corresponds to $100 \cdot (0.177 + 0.036) \approx 21\%$, where 0.177 is the coefficient of the intercept and 0.036 is the coefficient of Norway spruce.
- The effect of European beech on the median of LMF ($\tau = 0.5$) corresponds to $100 \cdot (0.177 - 0.041) \approx 14\%$, where 0.177 is the coefficient of the intercept and -0.041 is the coefficient of European beech.
